# Atomic force microscopy measurements of bacterial adhesion and biofilm formation onto clay-sized particles

**DOI:** 10.1038/srep16857

**Published:** 2015-11-20

**Authors:** Qiaoyun Huang, Huayong Wu, Peng Cai, Jeremy B. Fein, Wenli Chen

**Affiliations:** 1 State Key Laboratory of Agricultural Microbiology, Huazhong Agricultural University, Wuhan 430070, China; 2 Key Laboratory of Arable Land Conservation (Middle and Lower Reaches of Yangtze River), Ministry of Agriculture, College of Resources and Environment, Huazhong Agricultural University, Wuhan 430070, China; 3 University of Notre Dame, Department of Civil and Environmental Engineering and Earth Sciences, Notre Dame, IN 46556, USA

## Abstract

Bacterial adhesion onto mineral surfaces and subsequent biofilm formation play key roles in aggregate stability, mineral weathering, and the fate of contaminants in soils. However, the mechanisms of bacteria-mineral interactions are not fully understood. Atomic force microscopy (AFM) was used to determine the adhesion forces between bacteria and goethite in water and to gain insight into the nanoscale surface morphology of the bacteria-mineral aggregates and biofilms formed on clay-sized minerals. This study yields direct evidence of a range of different association mechanisms between bacteria and minerals. All strains studied adhered predominantly to the edge surfaces of kaolinite rather than to the basal surfaces. Bacteria rarely formed aggregates with montmorillonite, but were more tightly adsorbed onto goethite surfaces. This study reports the first measured interaction force between bacteria and a clay surface, and the approach curves exhibited jump-in events with attractive forces of 97 ± 34 pN between *E. coli* and goethite. Bond strengthening between them occurred within 4 s to the maximum adhesion forces and energies of −3.0 ± 0.4 nN and −330 ± 43 aJ (10^−18^ J), respectively. Under the conditions studied, bacteria tended to form more extensive biofilms on minerals under low rather than high nutrient conditions.

Soil bacteria live in an environment dominated by particle surfaces, and bacterial adhesion and subsequent biofilm formation of liquid-mineral interfaces are ubiquitous processes^1^. Bacterial colonization at the interfaces plays a critical role in the formation and stability of soil aggregates^2^,^3^, the weathering of minerals^4^, the control of bacterial activities^5^, the degradation and sequestration of organic carbon^6^, and the fate of pollutants^7^.

Bacterial adhesion onto solid surfaces is the initial step in biofilm formation, which is governed not only by the surface properties of the bacteria and the solids, but also by solution chemistry^8^,^9^,^10^. Over the past decades, bacterial adhesion to primary minerals and engineered surfaces has been extensively studied^11^,^12^. The solid surfaces that bacteria encounter in soils and sediments include primary minerals, clay minerals, iron oxides, and organic matter. Clay minerals and iron oxides are the most active inorganic colloid constituents in soils and sediments^1^. However, quantifying the extent of bacterial adhesion onto clay-size particles has been particularly challenging due to the difficulties associated with physical separation of bacteria from the particles, both of which are micron-scale objects of similar size.

Until recently, bacterial adhesion onto soil clay-sized particles has been investigated using density-gradient centrifugation approaches^8^,^13^,^14^,^15^, isothermal titration calorimetry^16^, parallel plate flow systems^17^, fluorescence microscopy together with a bacterial viability stain^10^, or attenuated total reflection-Flourier transformed infrared spectroscopy^18^. Using these methods, the maximum amounts of *Pseudomonas putida* cells adsorbed by montmorillonite, kaolinite and goethite were observed to be 3.2, 4.1 and 4.8 × 10^10^ cells g^−1^ in 10 mM Tris-HCl buffer (pH 7.0), respectively^8^. Similarly, *Escherichia coli* adhere to a greater extent onto kaolinite than onto montmorillonite in 0.1 ~ 100 mM KCl solutions, an observation that was suggested to be due to the chemical heterogeneity of kaolinite with anisotropic properties in the basal and edge surfaces^17^. The enthalpy changes of *Bacillus subtilis* adhesion to the above clay-sized particles ranged from −33 to −147 kJ kg^−1^ in 1 ~ 100 mM KNO_3_ solutions^16^. The removal of extracellular polymer substances from *B. subtilis* cells can reduce adhesion to clay minerals but enhance adhesion to goethite^19^. In contrast to mid-exponential phase, stationary-phase cells of *P. putida* exhibited a higher adhesion density on kaolinite, which was probably due to their smaller cell size and less negative surface charges^10^. Although a sizable body of literature has described bacterial adhesion onto clay-sized particles, direct measurements in adhesion patterns and forces at the single-cell level and direct evidence of cell adhesion to the kaolinite edge face are still lacking.

Atomic force microscopy (AFM) has been recently established as a powerful technique for imaging the surfaces of clay minerals^20^ or microbial cells^21^ and probing the forces driving cell-mineral adhesion^22^,^23^,^24^. Therefore, AFM allows for direct nanoscale observation on the surface morphologies of a bacterium aggregated with clay-sized particles and bacterial biofilms formed on these particles. Advanced imaging techniques including electron microscopy, confocal laser scanning microscopy with fluorescent labeling, magnetic resonance imaging, scanning transmission X-ray microscopy have been used for the direct visualization of bacteria-mineral aggregates and biofilms^25^,^26^. However, AFM represents a complementary technique by providing nanoscale surface characterization of bacteria-mineral associates and biofilms grown on minerals with minimal pretreatment and provides a better reflection of how they adhere to each other in natural settings. The surface morphology at nanoscale will enable a more important understanding of bacteria-mineral interactions and permit greater insight into the processes of bacterial surface colonization, but these studies are still rare.

In recent years, AFM has been used to quantify the forces guiding bacterial adhesion onto large (>~1 cm) primary minerals including muscovite, goethite, graphite^24^,^27^, magnetite, hematite^28^, chalcopyrite^29^,^30^, and pyrite^31^. For example, the retraction forces of *Shewanella oneidensis* from the (010) single crystal face of goethite were −0.80 ± 0.15 nN and −0.25 ± 0.10 nN after contact for 30 ~ 45 min under anaerobic and aerobic conditions, respectively^27^. Specific signatures in the retraction force curves under anaerobic conditions suggest that a 150-kDa putative iron reductase is mobilized within the outer membrane of *S. oneidensis* and specifically interacts with the goethite surface to facilitate the electron transfer process^27^. The retraction forces of *S. oneidensis* from single crystal growth faces of iron oxides were −1.1 ± 0.02 nN for the magnetite (111) face, −2.0 ± 0.04 nN for the magnetite (100) face, and −4.3 ± 0.04 nN for the hematite (001) face under anaerobic solutions^28^. In reality, primary minerals are very different from secondary clay-sized minerals in terms of particle size, specific surface area, surface charge properties which will lead to fundamental changes in forces controlling bacterial adhesion. In addition to prepare a high quality of bacterial probes, immobilization of clay-sized particles on substrates with a complete coverage and a low surface roughness in liquids is also needed to obtain reliable force spectroscopy between bacteria and clay-sized particles. Due to these difficulties, little information of direct measurements is known about the interaction forces of bacteria with clay-sized particles.

In the current study, three types of AFM measurements were performed: 1) nanoscale surface morphology of bacteria-mineral aggregates was determined; 2) force-distance curves for bacterial cells approaching and retracting clay-sized goethite particles were probed; and 3) the surface topographies of biofilms on clay-sized minerals were measured as a function of time and nutrient availability. The tested particles were kaolinite, montmorillonite and goethite, as representative minerals for 1:1 and 2:1 layer silicates and metal oxides, respectively, and each mineral type is a common soil constituent. Three Gram-negative strains and one Gram-positive strain were used in this study in order to determine whether the bacteria-mineral interactions are species specific or more generally applicable to a wider range of bacteria.

## Methods

### Minerals

A well-crystallized kaolinite (KGa-1b) was purchased from The Clay Minerals Society. Montmorillonite was obtained from Zhejiang Sanding Technology Co., Ltd (China). The montmorillonite colloids (<2 μm) were separated through sedimentation and were flocculated using CaCl_2_ (0.5 mol L^−1^) solution. The colloid suspension was repeatedly washed with deionized water (18.24 MΩ ∙ cm) and ethanol until the electrical conductivity was below 10 μS cm^−1^. The prepared colloids were oven-dried at 60 °C and sieved through a 0.149 mm mesh. Goethite was synthesized according to the method of Schwertmann and Cornell^32^. Mineral suspensions with concentrations of 1 and 3.3 g L^−1^ were prepared using deionized water. The clay mineral suspensions were disaggregated using a sonic dismembrator (Branson Sonifier 450) for 10 min at ~160 W. The goethite suspension was dispersed in an ultrasonic bath for 5 min.

### Bacteria

Four different soil bacterial strains were selected in this study, including Gram-negative strains *Escherichia coli* TG1, *Pseudomonas putida* KT2440, and *Agrobacterium tumefaciens* EHA105, and a Gram-positive strain *Bacillus subtilis* 168. *Escherichia coli* TG1 (CCTCC AB209135) and *Bacillus subtilis* 168 (CCTCC AB92082) were obtained from China Center for Type Culture Collection. *Pseudomonas putida* KT2440 (ATCC 47054) and *Agrobacterium tumefaciens* EHA105 (CGMCC 4821) were obtained from American Type Culture Collection and China General Microbiological Culture Collection Center, respectively. The strain of EHA105 was stored on YEB agar plates and the other strains were stored on Luria-Bertani (LB) agar plates at 4 °C. Single colonies of each strain were picked up and inoculated in 50 ml of Erlenmeyer flasks containing 10 ml of liquid medium. All cultures were grown aerobically on a rotary shaker (180 rpm) for 14 h and were harvested at mid-exponential growth phase. *E. coli* was grown in LB medium at 37 °C. *P. putida* and *B. subtilis* were cultured in LB at 28 °C. *A. tumefaciens* was incubated in YEB medium at 28 °C. The cells were pelleted by centrifugation for 10 min at 4100 g and 10 °C. The growth medium was decanted, and the pellet was rinsed three times by deionized water with pH of 5.6 ~ 5.9. The resulting pellets were resuspended in deionized water. The colony forming units per milliliter (CFU mL^−1^) was measured by a dilution-spread-plate method. The obtained bacterial suspensions were further used for preparation of bacteria-mineral aggregates and AFM cell probes. Similarly, a single colony was inoculated into 10 mL M9, LB or YEB medium and incubated until reaching mid-exponential growth phase. These cultures were applied for the experiment of bioflim formation. The compositions of each medium are listed as follows. LB medium (pH 7.0) per liter contains 5.0 g yeast extract, 10.0 g tryptone and 10.0 g NaCl. YEB medium (pH 7.0) per liter consists of 5.0 g beef extract, 1.0 g yeast extract, 5.0 g peptone, 5.0 g sucrose, and 0.5 g MgSO_4 _∙ 7H_2_O. M9 medium (pH 7.0) contains 48 mM Na_2_HPO_4_, 22 mM KH_2_PO_4_, 9 mM NaCl, 19 mM NH_4_Cl, 2 mM MgSO_4_, 0.1 mM CaCl_2_, and 8 mM glucose.

### Preparation of bacteria-mineral aggregates

Bacterial suspensions and ultrasonically dispersed mineral suspensions were mixed together to give a final bacterial concentration of 10^9^ cells mL^−1^ and a mineral concentration of 3 g L^−1^ in 10-mL centrifuge tubes. The bacteria-mineral suspensions were then stirred on a rotating rack end-over-end 40 times min^−1^ for 2 h at 25 °C. The adhesion of bacteria onto the mineral surfaces was expected to reach equilibrium within 2 h^9^.

### Biofilm formation on mineral surfaces

Minerals coated round glass coverslips were prepared as follows. Prior to coating, the coverslips (diameter 15 mm) were treated with 7:3 (v/v) H_2_SO_4_:H_2_O_2_ solution for 1 h, followed by rinsing with deionized water and sonication for 15 min in an ultrasonic cleaning bath. The coverslips were then immersed in ethanol for 15 min followed by washing with deionized water and sonication for 15 min. The washed coverslips were dried at 60 °C and stored in a desiccator. After cleaning, 0.4 mL of the ultrasound pre-treated mineral suspensions (1 g L^−1^) were pipetted onto the coverslips and left for 20 min to boil the minerals onto the glass substrate at ~120 °C. The coverslips were then removed to cool, rinsed continuously with deionized water for 20 s and dried at 60 °C. The mineral-coated and uncoated coverslips were autoclaved for 20 min at 121 °C. These coverslips were then placed in sterile polystyrene 6-well plates (Costar, Corning Incorporated, Corning, NY) and 0.25 mL of bacterial suspension was added on the coverslips. After bacterial adhesion for 10 min, 4.75 mL M9, LB or YEB medium was added to each well. The 6-well plates were statically positioned in a biochemical incubator in the dark at 37 °C for *E. coli* and 28 °C for the other strains. Coverslips were removed at certain intervals for up to 3 days. The biofilms on the mineral-coated and uncoated surfaces were rinsed gently in deionized water at least three times. The coverslips were dried by soaking liquid off of the edge with a paper towel and putting the coverslips in clean 6-well plates for 2 h.

### Morphology measurements of bacteria-mineral aggregates and biofilms

All morphology measurements were performed in air at room temperature (25 °C) using a MultiMode 8 AFM with a NanoScope V controller (Bruker). Two types of scanning modes were included based on the samples. One was the ScanAsyst mode using ScanAsyst-Air cantilevers with 0.4 N m^−1^ nominal spring constant (Bruker), and the other one was the tapping mode using RTESP cantilevers with 40 N m^−1^ nominal spring constant (Bruker). Bacteria-mineral aggregates were immobilized by drying on a mica surface. 10 μL aliquots of the experimental suspensions diluted by 30-fold using deionized water were dropped on the freshly cleaved mica and left undisturbed for 5 min to promote adhesion. The mica was then gently rinsed three times in deionized water and the samples were dried in air at 25 °C for 2 h. The mica was attached to a steel sample puck using a small piece of double-faced adhesive tape, and then was transferred into the sample stage on the AFM. To image the biofilms, the mineral-coated coverslips were also stuck to the sample puck in a similar fashion.

### AFM adhesion force measurements

Goethite was fixed into the surface of a thermoplastic adhesive called Tempfix (Electron Microscopy Sciences) as follows. A thin slice of Tempfix was placed on a square aluminum sheet (10 × 10 × 0.25 mm) and heated at ~120 °C to melt the adhesive. The Tempfix was then cooled to room temperature. 50 μL aliquots of ultrasound dispersed goethite suspensions (1 g L^−1^) were placed on the Tempfix-covered sheet, and quickly dried in a vacuum desiccator. The samples were then heated to ~38 °C, and then removed to cool. This allowed the Tempfix to become slightly sticky, while preventing the minerals from sinking too deeply into the polymer. The goethite-coated Tempfix was attached to a steel sample puck using a small piece of double-faced adhesive tape, and then was transferred into the AFM liquid cell. *E. coli* cells from suspensions were immobilized on triangular-shaped tipless AFM cantilevers (MLCT-O10 cantilevers with 0.05 N m^−1^ nominal spring constant, Bruker). To obtain better immobilization, cantilevers were washed individually with deioinzed water, ethanol, acetone and deioinzed water for 5 min and then dried in air. The rinsed cantilevers were first immersed in a drop of 0.01% (w/v) poly-L-lysine (MW 70 000 ~ 150 000, Sigma) for 1 min to create a positive charge on its surface with a micromanipulator (Eppendorf TransferMan NK2) mounted on an inverted optical microscope (Olympus IX 71). Subsequently, the cantilever was dried in air for 2 min using the micromanipulator, and then dipped into a drop of bacterial suspension (10^10^ cells mL^−1^) for 1 min to allow bacterial adhesion^33^. Each prepared bacterial probe was used immediately. All AFM force measurements were performed in a PicoForce scanning probe microscope with a NanoScope V controller (Bruker) in the contact mode at room temperature (25 °C) in deionized water, at a scan rate of 0.5 Hz, a ramp size of 1 um, and a trig threshold (contact force) of 1 nN. The surface contact times from 0 s to 20 s were set in order to reveal possible bond-strengthening. Scanning electron microscopy (JSM-6390LV, JEOL, Japan) was regularly used to confirm the integrity of the bacterial probe after measurements. About 20 force-distance curves were recorded that comprised a total of three different bacterial probes from three independent *E. coli* bacterial cultures. The spring constant of each cantilever was experimentally determined using the thermal tuning method^34^, yielding an average spring constant value of 0.074 ± 0.005 N m^−1^. Some models for analyzing the force-distance curves are presented as follows.

The maximum adhesion force F(t) or the adhesion energy E(t) were plotted as a function of the surface contact time (t) and fitted to the equations^33^:


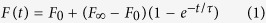



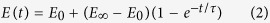


with F_0_ and E_0_ being the maximum adhesion force and the adhesion energy at 0 s contact time, F_∞_ and E_∞_ being the maximum adhesion force and the adhesion energy after bond strengthening, and τ being the characteristic time needed for the adhesion force or energy to strengthen.

The wormlike chain (WLC) model which describes the elasticity of flexible biopolymers was applied to analyze the multiple adhesion events in the retraction curves^35^. The force F(D) required to stretch a WLC chain to a length D is given by


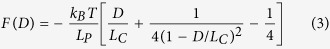


where k_B_ is the Boltzmann constant (1.38 × 10^−23^ J K^−1^), T is the absolute temperature (298 K), L_P_ is the persistence length, L_C_ is the biopolymer contour length taken as the total length of the polymer chain.

### AFM surface roughness determinations

Surface roughness of the goethite surface immobilized on Tempfix was measured using AFM in the ScanAsyst mode with ScanAsyst-Fluid cantilevers with 0.4 N m^−1^ nominal spring constant (Bruker) in deionized water. The goethite surface was imaged at five randomly chosen positions and surface plots were made to provide a three-dimensional perspective of the surface, from which the average roughness (R_a_) and root-mean-square (RMS) roughness (R_q_) were calculated. The R_a_ is the average deviation of the height values from the mean line/plane, and similarly the R_q_ is the root-mean-square deviation from the mean/plane, i.e. the standard deviation from the mean.

### Calculation of bacteria-mineral interaction energy profiles

Derjaguin-Landau-Verwey-Overbeek (DLVO) theory was used to calculate the interaction energies between the bacteria and mineral surfaces as a function of separation distance. Details of the total interaction energy calculations are given in the Supporting Information (SI).

## Results and Discussion

### Morphology of bacteria

**Figure 1:**
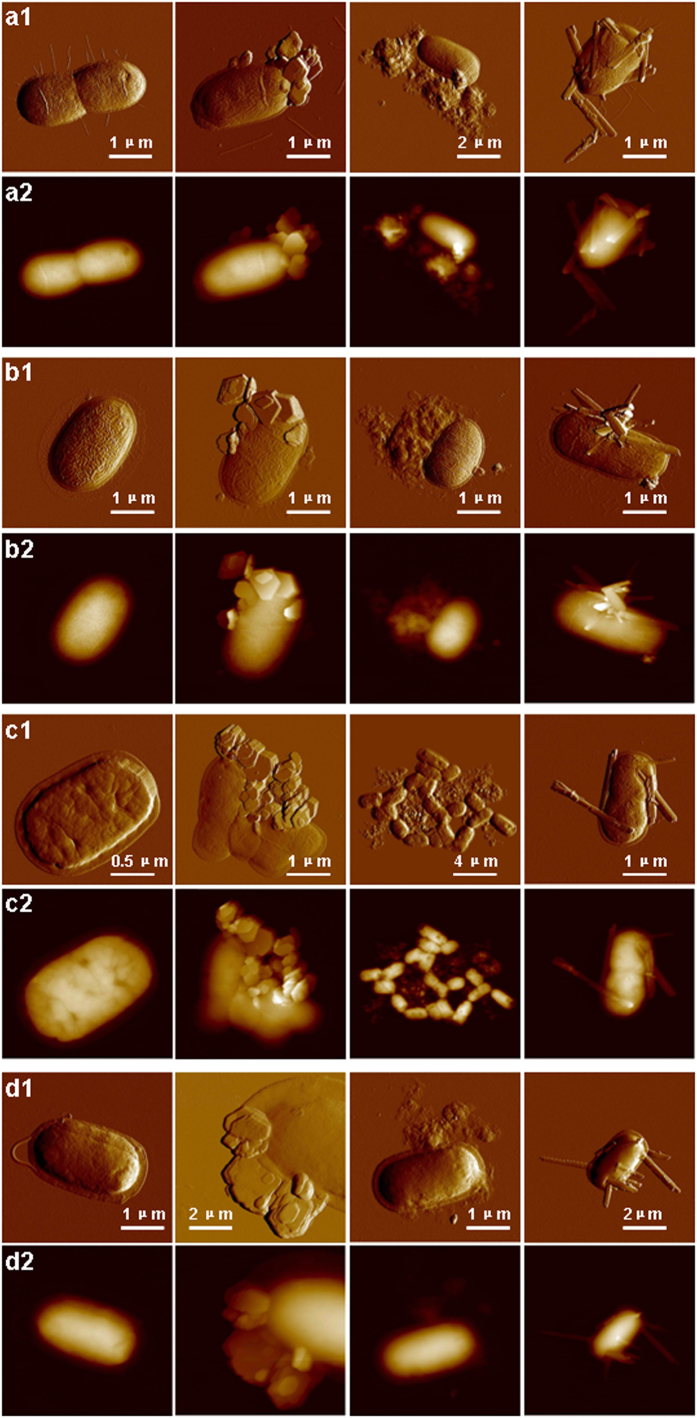
AFM images of bacteria and their aggregates with minerals in air. Peak force error and height images of *E. coli* (**a1**,**a2**), *P. putida* (**b1**,**b2**), *A. tumefaciens* (**c1**,**c2**), *B. subtilis* (**d1**,**d2**) and their aggregates with kaolinite (the second column), montmorillonite (the third column), and goethite (the fourth column).

Figure 1 shows representative peak force error and height images of bacteria and their associations with minerals. For bacteria, several observations can be made from these images. Firstly, height measurements (n = 40) of *B. subtilis* as an example yielded a cell length and width of 3.0 ± 0.7 μm and 1.2 ± 0.1 μm, respectively (Table 1). However, the cell height was only 0.28 ± 0.02 μm, which was much less than the expected cell diameter (~1 μm). An effect is attributed to the cell collapsing upon dehydration, which has already been reported for other bacterial species^36^,^37^,^38^. Secondly, wrinkles surrounding the cell surfaces were observed for Gram-negative bacteria, while relatively smooth cell surfaces were shown for Gram-positive bacteria after dehydration. This is probably ascribed to the different compositions of cell walls. The cytoplasmic membrane of Gram-negative bacteria is covered by a thin peptidoglycan layer overlayed by an asymmetrical bilayer of phospholipids and lipopolysaccharides, while the cytoplasmic membrane of Gram-positive bacteria is surrounded by a thick layer of peptidoglycan grafted with proteins and glycopolymers^39^,^40^. Thirdly, the drying process interestingly gave rise to flattened structures surrounding the cells. Since these features were 41 ± 6 nm or much thinner, it is very likely that they represent collapsed cell envelopes^40^. Lastly, the filaments are considered as pili with a thickness of 4.8 ± 0.8 nm for *E. coli* and flagella with a thickness of 9.2 ± 1.1 nm for *B. subtilis*. The cell walls of *P. putida* were covered by additional surface layers which were likely to be polysaccharide capsules with a thickness of 2.4 ± 0.3 nm. This form of bacterial polysaccharide capsules has been observed for *Zunongwangia profunda* by AFM^41^.

### Morphology of bacteria-mineral aggregates

As shown in Fig. 1, AFM images provide direct evidence at a single-cell level that all strains adhered predominantly to the edge surfaces of the kaolinite rather than to the basal surfaces. It is hard to find the aggregates of bacterial cells with montmorillonite and it appears that montmorillonite was weakly aggregated with bacterial cells. Scanning electron microscope (SEM) images from a previous study failed to clearly differentiate between bacterial cells and the edge and basal planes of kaolinite or montmorillonite in the aggregates^25^. Our high-resolution AFM images provide compelling evidence of cell adhesion onto kaolinite edge surfaces predominantly. The AFM images seem to indicate that goethite was closely adsorbed to bacterial cell surfaces. A previous study of bacterial-goethite particle aggregation using transmission electron microscopy (TEM) yielded only the alignment of the goethite crystals with the long axes of the cells likely due to sample preparation artifacts that were caused by centrifugation^42^. Centrifugation is not needed for AFM sample preparation, and hence the aggregation behavior that we describe is a better reflection of how these components interact in natural settings. The bacteria-mineral aggregates in Fig. 1 are expected to be formed in bacteria-mineral suspensions rather than in the processes of drying on mica surfaces. It is unlikely that evaporation-driven concentration would preferentially lead to aggregation via edge sites.

### Interaction energy between bacteria and minerals

**Figure 2:**
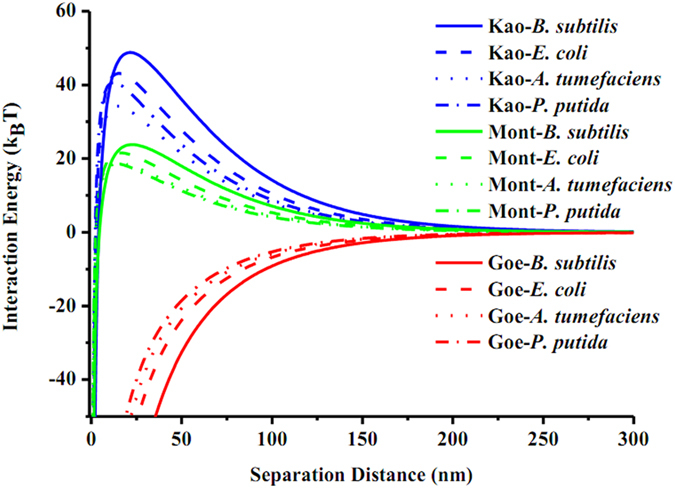
Total interaction energy profiles as a function of separation distance between bacteria and minerals in deionized water.

To understand the driving force responsible for bacteria-mineral adhesion, DLVO theory calculations and AFM adhesion force measurements were conducted. The DLVO interaction energy profiles are illustrated in Fig. 2. No energy barrier exists for bacteria-goethite interactions, indicating favorable interacting conditions for cell adhesion to goethite. The predicted irreversible adhesion in the primary energy minimum seems to be consistent with the close association between bacteria and goethite in the morphological results. The interaction energy profiles show energy barriers for bacterial adhesion to kaolinite (34 ~ 49 k_B_T) and montmorillonite (19 ~ 24 k_B_T) and no secondary energy minima (Table S1). The heights of the energy barriers for *B. subtilis* and *E. coli* adhesion to the clay minerals are greater than those measured for *P. putida* or *A. tumefaciens*, suggesting that adhesion of the clay minerals onto *B. subtilis* and *E. coli* is more unfavorable than onto the other strains.

Motile bacteria possess a kinetic energy that usually does not exceed 1 ~ 1.5 k_B_T as well as a typical thermal energy of approximately 0.5 ~ 1.5 k_B_T^43^,^44^. Therefore, for adhesion to occur, the energy barrier can not be significantly higher than approximately 3 k_B_T. However, this study and previous studies document extensive adhesion under conditions where DLVO theory predicts energy barriers to adhesion greater than 3 k_B_T^10^. The discrepancies between DLVO predictions and observations suggest either limitations to the DLVO theory or that other pathways exist to overcome the energy barriers. The limitation of the DLVO model may be due to its assumption of smooth and uniform surface charges on each of the interacting objects. The clay minerals used in this study exhibit chemical heterogeneity on their basal and edge surfaces^45^. Kaolinite consists of one tetrahedral sheet and one octahedral sheet, and the silica tetrahedra carry a small permanent negative charge due to isomorphic substitution of Al^3+^ for Si^4+^. Similarly, the octahedral and the edge surfaces of kaolinite carry variable charge depending on the pH of the system^45^,^46^. Montmorillonite is composed of an octahedral sheet sandwiched between two tetrahedral sheets, each of which possesses large permanent negative charge and negligible variable charge^45^,^47^. The silica surface of kaolinite is considered to be negatively charged at pH > 4, whereas the alumina surface of kaolinite is negatively charged at pH > 8 and positively charged at pH < 6^48^. Therefore, under our experimental conditions (pH 5.6 ~ 5.9), the two basal planes of montmorillonite are negatively charged, and the octahedral and edge surfaces of kaolinite are slightly positively charged. Positive charge from the exposed edge surfaces of kaolinite may help the mineral adhere to bacteria in the primary energy minimum. This inference is in good agreement with our AFM images that show bacterial cells predominantly adhered to the edge surfaces of kaolinite rather than to the basal surfaces.

Besides chemical heterogeneities of kaolinite that could contribute to inconsistencies with DLVO predictions, other non-DLVO factors such as polymer bridging, surface roughness and Lewis acid-base interactions are also probably responsible for the aggregation of bacteria-clay minerals. Biopolymers are often found to extend out from the cell surfaces into solutions over distances of up to 100 nm^49^. The biopolymers can probably penetrate the energy barriers to enhance cell adhesion onto clay minerals, when considering that the small radii of biopolymers are expected to have relatively low energy barriers^11^. Furthermore, interaction energy profiles are intensively affected by surface roughness^50^, and the irregular surface of clay minerals probably leads to a complex distribution of interaction energies. The heterogeneity in the interaction energy profiles may result in association of cells with clay minerals. If polymer bridging and surface roughness help bacterial cells conquer the energy barriers, Lewis acid-base interactions become to favor irreversible cell adhesion. Taken together, kaolinite with the main help of chemical heterogeneity forms a more stable aggregate with bacteria, whereas montmorillonite is loosely aggregated with bacteria.

### Force-distance curves of E. coli with goethite

**Figure 3:**
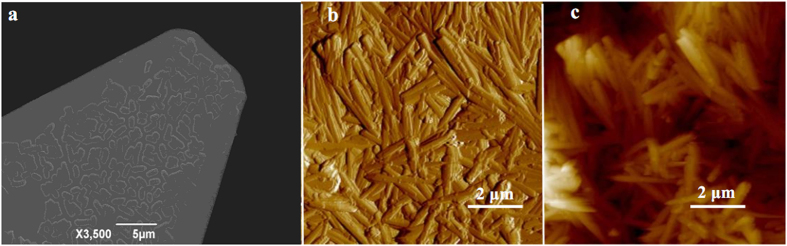
SEM image of a tipless cantilever with immobilized bacteria (**a**) and AFM peak force error (**b**) and height (**c**) images of goethite fixed to a Tempfix surface in deionized water.

The bacteria-mineral pair of *E. coli* and goethite was selected for the adhesion force measurements. To maintain consistent contact with the goethite surface, we insured complete coverage of the tipless cantilever with bacterial cells (Fig. 3a). The goethite was immobilized on the aluminum sheet using Tempfix and completely coated the substratum (Fig. 3b,c). The average roughness (R_a_) and root-mean-square roughness (R_q_) of the goethite surface in deionized water were determined to be 142 ± 32 nm and 113 ± 27 nm, respectively. We chose to use this complete coverage approach rather than a single-cell approach to determine the interaction forces in order to avoid possible interference introduced from forces between the uncoated portion of the cantilever and the goethite surface.

**Figure 4:**
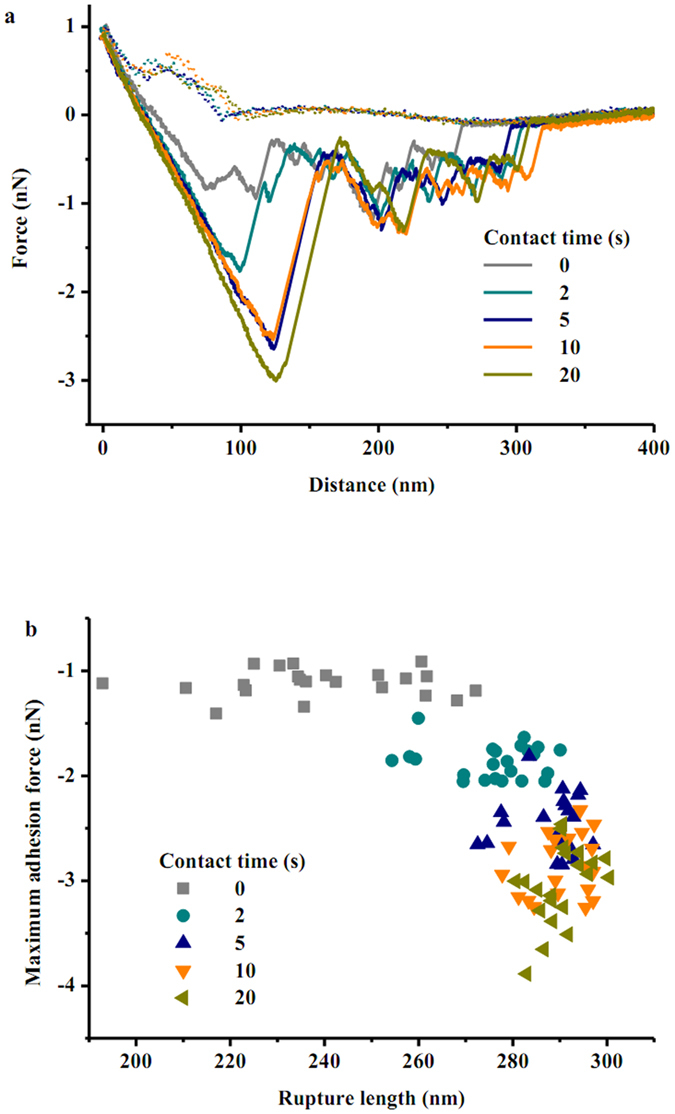
(**a**) Representative force-distance curves between *E. coli* and goethite as a function of the surface contact time in water. For each approach-retraction cycle, the upper dataset corresponds to approach values, whereas the lower dataset corresponds to retraction values. (**b**) The summary of the maximum adhesion forces and rupture lengths between *E. coli* and goethite in deionized water.

**Figure 5:**
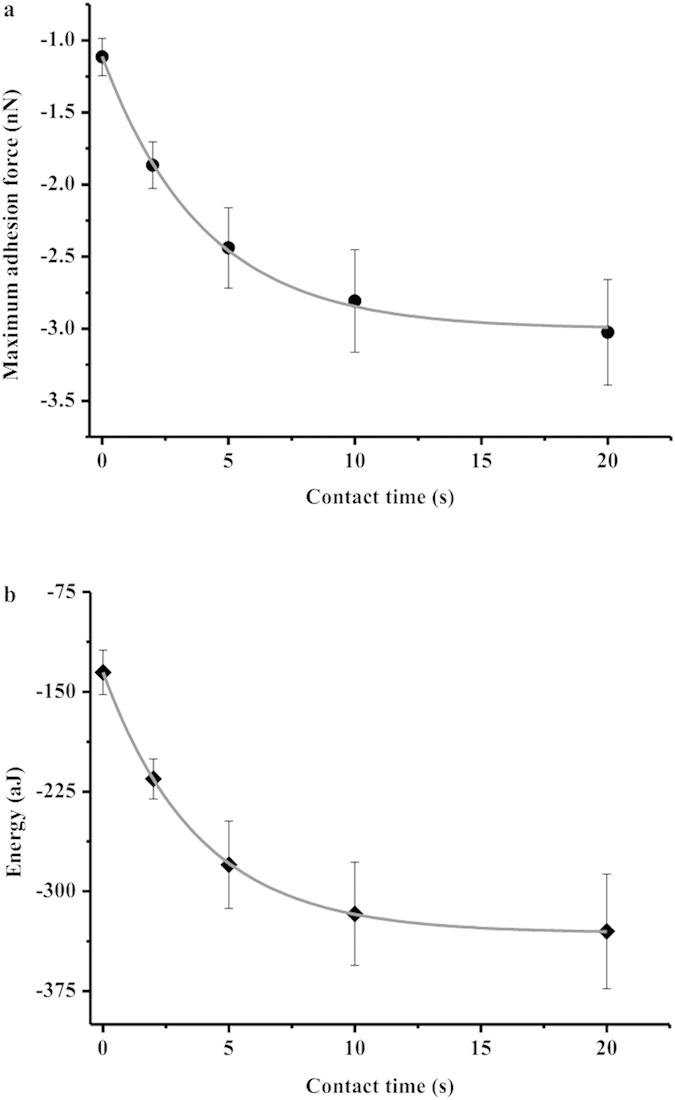
The maximum adhesion forces (**a**) and the adhesion energies (**b**) upon retraction of *E. coli* from goethite as a function of the surface contact time. Note that adhesion forces are in nanonewton and energy values are in attojoules (aJ = 10^−18^ J).

Representative force-distance curves between *E. coli* and goethite as a function of contact time in water are shown in Fig. 4a. To the best of our knowledge, this is the first study to report the measured adhesion forces between bacteria and clay-sized minerals in water. As the cell probe approaches the goethite, the approach curves exhibit jump-in events with attractive forces of 97 ± 34 pN (n = 108). This measured attractive force is consistent with DLVO theory which predicts no energy barrier between the cells and the goethite under the experimental conditions. Although several previous studies have reported theoretical energy-distance profiles for bacteria approaching a goethite surface based on DLVO calculations^17^,^51^, experimental measurements are scarce. After contact between the cells and the goethite, multiple adhesion events were observed during retraction, likely due to the effects of biomacromolecules from the cell surfaces that were adsorbed to the goethite. In the retraction curves, a wide range of rupture lengths was found, likely due to heterogeneities of cell wall biopolymers and surface roughness of the goethite. The rupture length is arbitrarily defined by the length from the contact point to the rupture point where the adhesion force becomes zero. The mean rupture length increases from 239 ± 20 nm to 290 ± 6 nm with decreasing maximum adhesion forces from −1.1 ± 0.1 nN to −3.0 ± 0.4 nN and adhesion energies from −135 ± 17 aJ (10^−18^ J) to −330 ± 43 aJ (Figs 4b and 5). Increasing contact time of the cell probe at the goethite surface clearly causes bond strengthening (Fig. 5). A fitted time of 4 s is needed for the adhesion force and energy to strengthen between the cells and the goethite. The maximum adhesion forces observed here were similar to those of *S. oneidensis* with single crystal growth faces of magnetite and hematite (−1.1 ~ −4.3 nN)^28^, and were larger than those between *S. oneidensis* and single crystals of goethite (−0.25 ~ −0.80 nN)^27^. The stronger interaction of bacteria with clay-sized goethite compared to single crystals of goethite is likely due to multiple contact points that probably exist between the bacterial cells and the surface of the clay-sized goethite. The observed adhesion energies vary from −32 900 ± 4 040 k_B_T to −80 300 ± 10 500 k_B_T with increasing contact time. An adhesion energy of approximately 10 k_B_T represents the border between reversible and irreversible adhesion^43^. Therefore, the large adhesion energies that we observed are sufficient for thousands of cells to bind irreversibly to goethite in the primary energy minimum. The large adhesion energies confirm the tight aggregates of bacteria with goethite. These experimentally measured strong attractions may explain observations from previous studies that bacterial adhesion to goethite is detrimental to cell survival^17^.

The WLC model of polymer elasticity was used to quantify the conformational properties of bacterial surface biopolymers in the sawtooth patterns of retraction curves. Representative retraction curves were fitted to the WLC model that describes the force needed to stretch a biopolymer chain to a certain length (Fig. S1). It should be noted that the WLC model can not be applied to all the retraction data because the WLC model is only applicable to the stretching of single molecule chains^35^. As the cells immobilized on the cantilever retract from the goethite, single molecules involved in the adhesion can be stretched occasionally. Peaks with large force magnitudes were assumed to be caused by pulling of more than one molecule, and thus were excluded from the WLC analysis. Stretching single molecules usually requires forces on the order of a few hundred pN^52^. If larger forces are observed, more than one chain may be pulled by the goethite surface. The persistence length of the biopolymer was estimated to be 0.154 nm, which is the same as the C-C bond length^53^, suggesting that the biopolymer chains interacting with goethite are very flexible. The contour lengths ranged between 182 nm and 347 nm, which can be used as an indication of the distance that biopolymers extend from the bacterial surface.

### Morphology of biofilm formation on minerals

**Figure 6:**
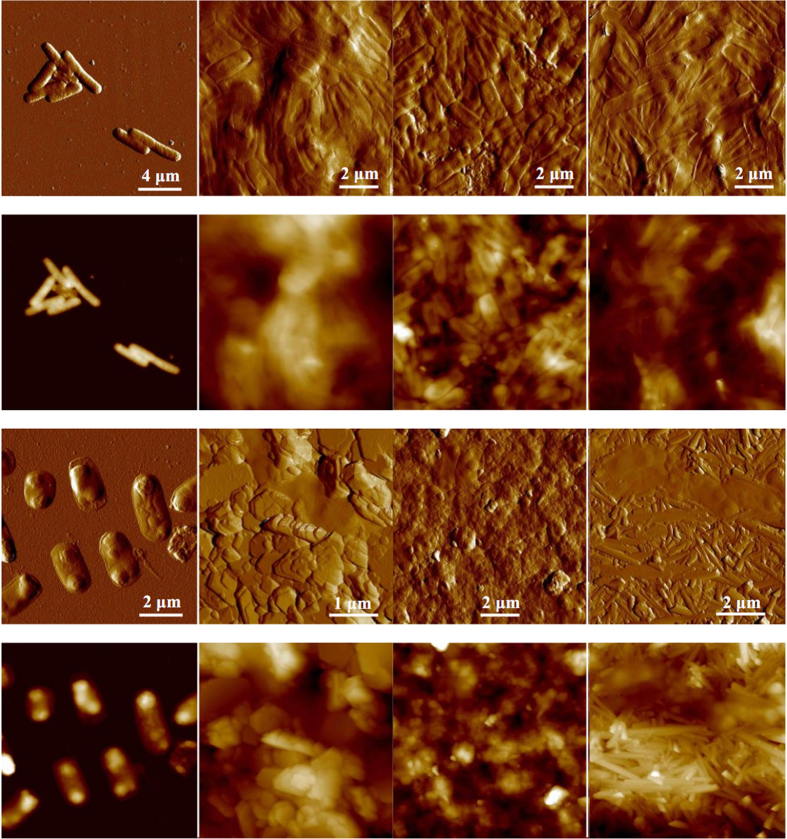
AFM peak force error and height images of *E. coli* biofilm formed on coverslips (the first column), kaolinite (the second column), montmorillonite (the third column) and goethite (the fourth column) surfaces after 2 days in M9 (the upper two rows) and LB medium (the lower two rows).

The effects of nutrient availability on the surface morphology of *E. coli* biofilm grown for 2 days on different clay-sized minerals are shown in Fig. 6. Dense cell layers were observed on minerals after growth in the M9 medium rather than the LB medium and the morphology of the biofilm surface was controlled by the height of the mineral surfaces. The images of the other bacterial biofilms grown over time are shown in Figs S2 to S7. For *P. putida* grown in both media, attached cells formed aggregates or colonies on solid surfaces at 10 min (Figs S2 and S3). The bacteria gradually formed a near-continuous layer on the mineral surfaces over time in the M9 medium. However, the bacteria covered virtually all exposed surfaces of the montmorillonite within 2 days in the LB medium, in contrast to the less extensive coverage observed for the kaolinite and goethite surfaces. For *A. tumefaciens* and *B. subtilis* grown in each medium tested, attached cells were individually distributed on the solid surfaces at 10 min (Figs S4 to S7). Flagella of *B. subtilis* grown in the LB medium were observed, and their presence may help cells adhere to the montmorillonite surface. *B. subtilis* formed macrocolonies or biofilms on the tested minerals in the M9 medium to a greater extent than was observed in the LB medium. Interestingly, spores of *B. subtilis* were observed to start forming on minerals during 1 ~ 2 days in the M9 medium, whereas no spores were present in the LB medium within 2 days. Generally, all examined strains tended to form more extensive biofilms on the tested surfaces under low nutrient conditions than were observed under the high nutrient conditions. Of the four bacterial species studied here, *E. coli* formed the most extensive biofilms on the minerals in this study.

## Additional Information

**How to cite this article**: Huang, Q. *et al.* Atomic force microscopy measurements of bacterial adhesion and biofilm formation onto clay-sized particles. *Sci. Rep.* **5**, 16857; doi: 10.1038/srep16857 (2015).

## Supplementary Material

Supplementary Information

## Figures and Tables

**Table 1:** Overview of AFM measurements performed on the four strains.

	*E. coli*	*P. putida*	*A. tumefaciens*	*B. subtilis*
Cell length/μm	2.3 ± 0.5	2.2 ± 0.4	1.9 ± 0.4	3.0 ± 0.7
Cell width/μm	1.3 ± 0.1	1.2 ± 0.1	1.1 ± 0.1	1.2 ± 0.1
Cell height/nm	237 ± 28	240 ± 26	184 ± 26	282 ± 21
Cell envelop thickness/nm	22 ± 4	24 ± 4	24 ± 5	41 ± 6
Flagella thickness/nm				9.2 ± 1.1
Pili thickness/nm	4.8 ± 0.8			
Polysaccharide capsule thickness/nm		2.4 ± 0.3		

AFM values were obtained from at least 40 measurements.

## References

[b1] Chenu C. & Stotzky G. Interactions between microorganisms and soil particles: an overview. In Interactions between Soil Particles and Microorganisms: Impact on the Terrestrial Ecosystem. Huang P. M., Bollag J. M. & Senesi N. (eds): John Wiley and Sons, USA pp. 3–40 (2002).

[b2] Young I. M. & Crawford J. W. Interactions and self-organization in the soil-microbe complex. Science 304, 1634–1637 (2004). 15192219 10.1126/science.1097394

[b3] Huang P. M., Wang M. K. & Chiu C. Y. Soil mineral–organic matter–microbe interactions: impacts on biogeochemical processes and biodiversity in soils. Pedobiologia 449, 609–635 (2005).

[b4] Kim J., Dong H., Seabaugh J., Newell S. W. & Eberl D. D. Role of microbes in the smectite-to-illite reaction. Science 303, 830–832 (2004). 14764877 10.1126/science.1093245

[b5] Wu H. *et al.* Soil colloids and minerals modulate metabolic activity of *Pseudomonas putida* measured using microcalorimetry. Geomicrobiol. J. 31, 590–596 (2014a).

[b6] Lützow M. V. *et al.* Stabilization of organic matter in temperate soils: mechanisms and their relevance under different soil conditions-a review. Eur. J. Soil Sci. 57, 426–445 (2006).

[b7] Morrow J. B., Stratton R., Yang H. H., Smets B. F. & Grasso D. Macro- and nanoscale observations of adhesive behavior for several *E. coli* strains (O157:H7 and environmental isolates) on mineral surfaces. Environ. Sci. Technol. 39, 6395–6404 (2005). 16190192 10.1021/es0500815

[b8] Jiang D., Huang Q., Cai P., Rong X. & Chen W. Adsorption of *Pseudomonas putida* on clay minerals and iron oxide. Colloids Surf. B 54, 217–221 (2007). 10.1016/j.colsurfb.2006.10.030 17142018

[b9] Wu H., Jiang D., Cai P., Rong X. & Huang Q. Effects of low-molecular-weight organic ligands and phosphate on adsorption of *Pseudomonas putida* by clay minerals and iron oxide. Colloids Surf. B 82, 147–151 (2011). 10.1016/j.colsurfb.2010.08.035 20843669

[b10] Wu H. *et al.* Adhesion of *Pseudomonas putida* onto kaolinite at different growth phases. Chem. Geol. 390, 1–8 (2014b).

[b11] Hori K. & Matsumoto S. Bacterial adhesion: from mechanism to control. Biochem. Eng. J. 48, 424–434 (2010).

[b12] Tuson H. H. & Weibel D. B. Bacteria-surface interactions. Soft. Mat. 9, 4368–4380 (2013). 10.1039/C3SM27705D PMC3733390 23930134

[b13] Rong X., Chen W., Huang Q., Cai P. & Liang W. *Pseudomonas putida* adhesion to goethite: studied by equilibrium adsorption, SEM, FTIR and ITC. Colloids Surf. B 80, 79–85 (2010). 10.1016/j.colsurfb.2010.05.037 20620892

[b14] Wu H. *et al.* Adsorption of *Pseudomonas putida* on soil particles size fractions: effects of solution chemistry and organic matter. J. Soils Sediments 12, 143–149 (2012).

[b15] Zhao W., Walker S. L., Huang Q. & Cai P. Adhesion of bacterial pathogens to soil colloidal particles: influences of cell type, natural organic matter, and solution chemistry. Water Res. 53, 35–46 (2014). 24495985 10.1016/j.watres.2014.01.009

[b16] Hong Z., Rong X., Cai P., Liang W. & Huang Q. Effects of temperature, pH and salt concentrations on the adsorption of *Bacillus subtilis* on soil clay minerals investigated by microcalorimetry. Geomicrobiol. J. 28, 686–691 (2011).

[b17] Cai P., Huang Q. & Walker S. L. Deposition and survival of *Escherichia coli* O157:H7 on clay minerals in a parallel plate flow system. Environ. Sci. Technol. 47, 1896–1903 (2013). 23346967 10.1021/es304686a

[b18] Wu H. *et al.* *In situ* ATR-FTIR study on the adhesion of *Pseudomonas putida* to Red soil colloids. J. Soils Sediments 14, 504–514 (2014c).

[b19] Hong Z. *et al.* The effect of extracellular polymeric substances on the adhesion of bacteria to clay minerals and goethite. Chem. Geol. 360–361, 118–125 (2013).

[b20] Bickmore B. R., Hochella M. F., Bosbach D. & Charlet L. Methods for performing atomic force microscopy imaging of clay minerals in aqueous solutions. Clay Clay Miner. 47, 573–581 (1999).

[b21] Dufrêne Y. F. Using nanotechniques to explore microbial surfaces. Nat. Rev. Microbiol. 2, 451–460 (2004). 15152201 10.1038/nrmicro905

[b22] Bowen W. R., Hilal N., Lovitt R. W. & Wright C. J. Direct measurements of the force of adhesion of a single biological cell using an atomic force microscope. Colloids Surf. A 136, 231–234 (1998).

[b23] Razatos A., Ong Y.-L., Sharma M. M. & Georgiou G. Molecular determinants of bacterial adhesion monitored by atomic force microscopy. Proc. Natl. Acad. Sci. USA 95, 11059–11064 (1998). 9736689 10.1073/pnas.95.19.11059 PMC21595

[b24] Lower S. K., Tadanier C. J. & Hochella M. F. Measuring interfacial and adhesion forces between bacteria and mineral surfaces with biological force microscopy. Geochim. Cosmochim. Acta 64, 3133–3139 (2000).

[b25] Rong X. *et al.* Interaction of *Pseudomonas putida* with kaolinite and montmorillonite: a combination study by equilibrium adsorption, ITC, SEM and FTIR. Colloids Surf. B 64, 49–55 (2008). 10.1016/j.colsurfb.2008.01.008 18282693

[b26] Neu T. R. *et al.* Advanced imaging techniques for assessment of structure, composition and function in biofilm systems. FEMS Microl. Ecol. 72, 1–21 (2010). 10.1111/j.1574-6941.2010.00837.x 20180852

[b27] Lower S. K., Hochella M. F. & Beveridge T. J. Bacterial recognition of mineral surfaces: nanoscale interactions between *Shewanella* and α-FeOOH. Science 292: 1360–1363 (2001). 11359008 10.1126/science.1059567

[b28] Neal A. L., Bank T. L., Hochella M. F. & Rosso K. M. Cell adhesion of *Shewanella oneidensis* to iron oxide minerals: effect of different single crystal faces. Geochem. T. 6, 77–84 (2005). 10.1186/1467-4866-6-77 PMC1475793 35430629

[b29] Zhu J. *et al.* Adhesion forces between cells of Acidithiobacillus ferrooxidans, Acidithiobacillus thiooxidans or Leptospirillum ferrooxidans and chalcopyrite. Colloids Surf. B 94, 95–100 (2012). 10.1016/j.colsurfb.2012.01.022 22341516

[b30] Diao M., Nguyen T. A. H., Taran E., Mahler S. & Nguyen A. V. Differences in adhesion of *A. thiooxidans* and *A. ferrooxidans* on chalcopyrite as revealed by atomic force microscopy with bacterial probes. Miner. Eng. 61, 9–15 (2014a).

[b31] Diao M., Taran E., Mahler S., Nguyen T. A. H. & Nguyen A. V. Quantifying adhesion of acidophilic bioleaching bacteria to silica and pyrite atomic force microscopy with a bacterial probe. Colloids Surf. B 115, 229–236 (2014b). 10.1016/j.colsurfb.2013.11.047 24355385

[b32] Schmertmann U. & Cornell R. M. Iron Oxides in the Laboratory: Preparation and Characterization. New York, USA: VCH (1991).

[b33] van der Mei H. C., Rustema-Abbing M., de Vries J. & Busscher H. J. Bond strengthening in oral bacterial adhesion to salivary conditioning films. Appl. Environ. Microbiol. 74, 5511–5515 (2008). 18641154 10.1128/AEM.01119-08 PMC2546621

[b34] Hutter J. L. & Bechhoefer J. Calibration of atomic-force microscope tips. Rev. Sci. Instrum. 64, 1868–1873 (1993).

[b35] Janshoff A., Neitzert M., Oberdörfer Y. & Fuchs H. Force spectroscopy of molecular systems-single molecule spectroscopy of polymers and biomolecules. Angew Chem. Int. Ed 39, 3212–3237 (2000). 10.1002/1521-3773(20000915)39:18<3212::aid-anie3212>3.0.co;2-x 11028062

[b36] Doktycz M. J. *et al.* AFM imaging of bacteria in liquid media immobilized on gelatin coated mica surfaces. Ultramicroscopy 97, 209–216 (2003). 12801673 10.1016/S0304-3991(03)00045-7

[b37] Beckmann M. A. *et al.* Measuring cell surface elasticity on enteroaggregative *Escherichia coli* wild type and dispersin mutant by AFM. Ultramicroscopy 106, 695–702 (2006). 16682120 10.1016/j.ultramic.2006.02.006

[b38] Gillis A., Dupres V., Delestrait G., Mahillon J. & Dufrêne Y. F. Nanoscale imaging of *Bacillus thuringiensis* flagella using atomic force microscopy. Nanoscale 4, 1585–1591 (2012). 22159046 10.1039/c1nr11161b

[b39] Silhavy T. J., Kahne D. & Walker S. The bacterial cell envelope. Cold Spring Harb. Perspect. Biol. 2, a000414 (2010). 20452953 10.1101/cshperspect.a000414 PMC2857177

[b40] Tripathi P. *et al.* Towards a nanoscale view of lactic acid bacteria. Micron 43, 1323–1330 (2012). 22293169 10.1016/j.micron.2012.01.001

[b41] Su H. *et al.* Characterization of bacterial polysaccharide capsules and detection in the presence of deliquescent water by atomic force microscopy. Appl. Environ. Microbiol. 78, 3476–3479 (2012). 22344657 10.1128/AEM.00207-12 PMC3346466

[b42] Glasauer S., Langley S. & Beveridge T. J. Sorption of Fe (hydr) oxides to the surface of *Shewanella putrefaciens*: cell-bound fine-grained minerals are not always formed De Novo. Appl. Environ. Microbiol. 67, 5544–5550 (2001). 11722905 10.1128/AEM.67.12.5544-5550.2001 PMC93342

[b43] van Loosdrecht M. C. M., Lyklema J., Norde W. & Zehnder A. J. B. Bacterial adhesion: a physicochemical approach. Microb. Ecol. 17, 1–15 (1989). 24197119 10.1007/BF02025589

[b44] Hahn M. W. & Ơ Melia C. R. Deposition and reentrainment of Brownian particles in porous media under unfavorable chemical conditions: some concepts and applications. Environ. Sci. Technol. 38, 210–220 (2004). 14740738 10.1021/es030416n

[b45] Sposito G. The Surface Chemistry of Soils. New York, USA: Oxford University Press (1984)

[b46] Tombácz E. & Szekeres M. Surface charge heterogeneity of kaolinite in aqueous suspension in comparison with montmorillonite. Appl. Clay Sci. 34, 105–124 (2006).

[b47] Tombácz E. & Szekeres M. Colloidal behavior of aqueous montmorillonite suspensions: the specific role of pH in the presence of indifferent electrolytes. Appl. Clay Sci. 27, 75–94 (2004).

[b48] Gupta V., Hampton M. A., Stokes J. R., Nguyen A. V. & Miller J. D. Particle interactions in kaolinite suspensions and corresponding aggregate structures. J Colloid Interf. Sci. 359, 95–103 (2011). 10.1016/j.jcis.2011.03.043 21489550

[b49] Rijnaarts H. H. M., Norde W., Bouwer E. J., Lyklema J. & Zehnder A. J. B. Reversibility and mechanism of bacterial adhesion. Colloids Surf. B 4, 5–22 (1994).

[b50] Bhattacharjee S., Ko C. H. & Elimelech M. DLVO interaction between rough surfaces. Langmuir 14, 3365–3375 (1998).

[b51] Hong Z. *et al.* Initial adhesion of *Bacillus subtilis* on soil minerals as related to their surface properties. Eur. J. Soil Sci. 63, 457–466 (2012).

[b52] Rief M., Gautel M., Oesterhelt F., Fernandez J. M. & Gaub H. E. Reversible unfolding of individual titin immunoglobulin domains by AFM. Science 276, 1109–1112 (1997). 9148804 10.1126/science.276.5315.1109

[b53] Park B. P. & Abu-Lail N. I. Variations in the nanomechanical properties of virulent and avirulent *Listeria monocytogenes*. Soft Mat. 6, 3898–3909 (2010). 10.1039/b927260g PMC2944262 20871743

