# Supplementary material for: Atomic force microscopy measurements of bacterial adhesion and biofilm formation onto clay-sized particles
Source: Sci Rep. 2015 Nov 20;5:16857. doi: 10.1038/srep16857 (PMC4653644; doi:10.1038/srep16857)
Supplement: Supplementary Information [file srep16857-s1.doc]

Supporting Information for

Atomic force microscopy measurements of bacterial adhesion and biofilm formation onto clay-sized particles

Qiaoyun Huang^a,b,*^, Huayong Wu^a,b,*^, Peng Cai^a,b^, Jeremy B. Fein^c^, and Wenli Chen^a,§^

^a^ State Key Laboratory of Agricultural Microbiology, Huazhong Agricultural University, Wuhan 430070, China

^b^ Key Laboratory of Arable Land Conservation (Middle and Lower Reaches of Yangtze River), Ministry of Agriculture, College of Resources and Environment, Huazhong Agricultural University, Wuhan 430070, China

^c^ University of Notre Dame, Department of Civil and Environmental Engineering and Earth Sciences, Notre Dame, IN 46556, USA

* These authors contributed equally to this work.

^§^Corresponding author: W. Chen, E-mail: [wlchen@mail.hzau.edu.cn](mailto:wlchen@mail.hzau.edu.cn). Tel: +86-27-87282730; Fax: +86-27-87280670. Correspondence and requests for materials should be addressed to W.C. (email: [wlchen@mail.hzau.edu.cn](mailto:wlchen@mail.hzau.edu.cn))

**Table S1.** Electrokinetic potentials and hydrodynamic radii of bacteria and minerals as well as the heights of the energy barrier determined from DLVO theory calculation in deionized water.

| Bacteria/Minerals | Zeta potential  (mV) | Hydrodynamic  radius (nm) | Energy barrier height (k_B_T) for bacteria-mineral interaction | | | | |
| --- | --- | --- | --- | --- | --- | --- | --- |
|  |  |  | *Bacteria-*  *Kaolinite* |  | *Bacteria-*  *Montmorillonite* |  | *Bacteria-*  *Goethite* |
| *E. coli* | -50.53 ± 0.90 | 2025 ± 34 | 43.14 |  | 21.57 |  | No energy barrier |
| *P. putida* | -40.40 ± 0.70 | 1576 ± 50 | 40.60 |  | 18.64 |  | No energy barrier |
| *A. tumefaciens* | -47.50 ± 0.80 | 1049 ± 25 | 34.24 |  | 18.77 |  | No energy barrier |
| *B. subtilis* | -66.57 ± 3.30 | 2453 ± 19 | 48.81 |  | 23.80 |  | No energy barrier |
| Kaolinite | -24.50 ± 0.61 | 652 ± 55 |  |  |  |  |  |
| Montmorillonite | -23.63 ± 0.49 | 302 ± 1 |  |  |  |  |  |
| Goethite | 36.40 ± 1.80 | 207 ± 4 |  |  |  |  |  |

**Fig. S1.** Representative retraction curves between *E. coli* and goethite in deionized water with contact times of 0 s (a), 2 s (b), 5 s (c), 10 s (d), and 20 s (e). Gray curves correspond to the wormlike chain (WLC) model fitting.


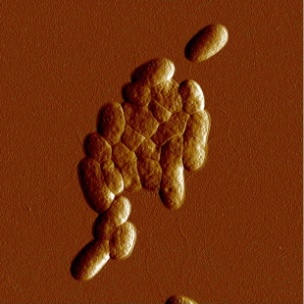

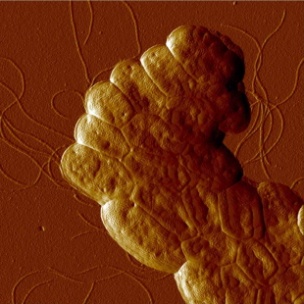

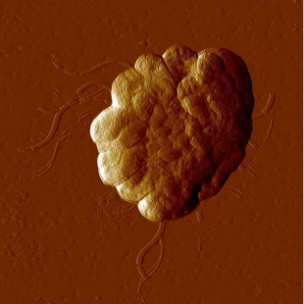

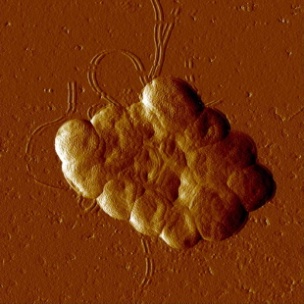


**Coverslips**

10 min

12 h

24 h

48 h

**2 μm**

**4 μm**

**2 μm**

**2 μm**


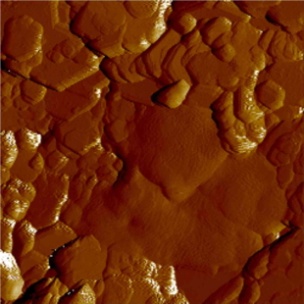

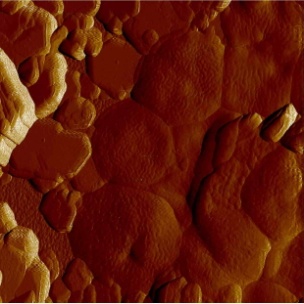


24 h

48 h

**Kaolinite**

**1 μm**

**1 μm**


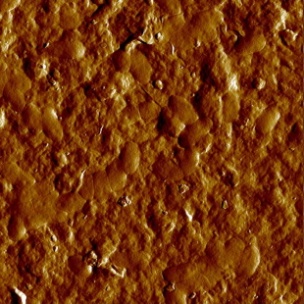

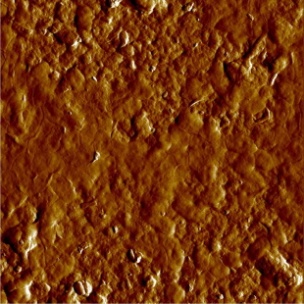

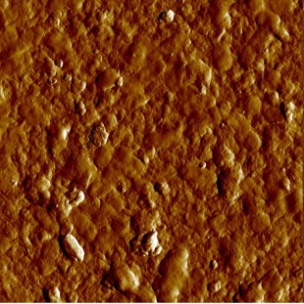

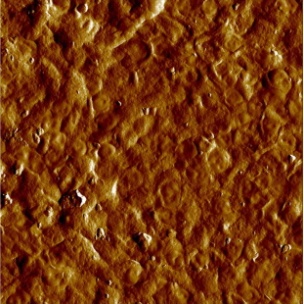


**Montmorillonite**

10 min

12 h

24 h

48 h

**5 μm**

**5 μm**

**5 μm**

**5 μm**


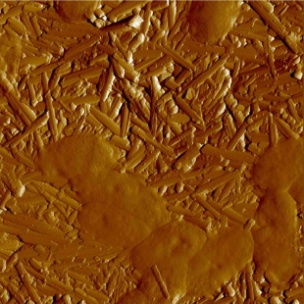

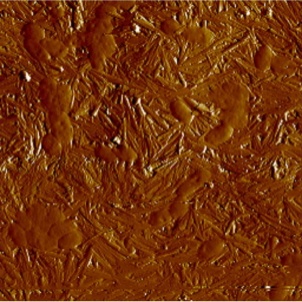

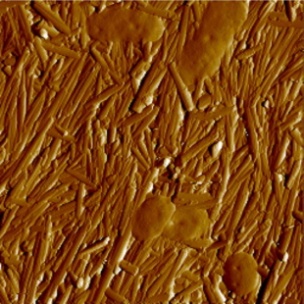

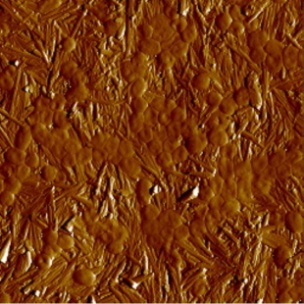


10 min

12 h

24 h

48 h

**Goethite**

**2 μm**

**2 μm**

**5 μm**

**5 μm**

**Fig. S2.** AFM peak force error images of *P. putida* biofilms in M9 medium.


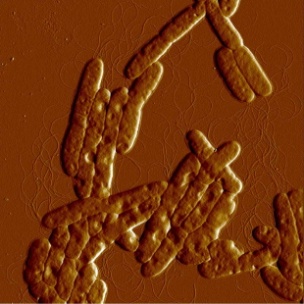

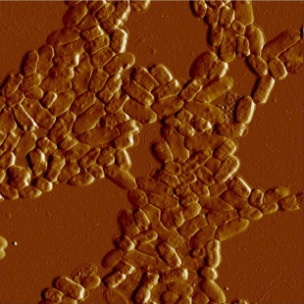

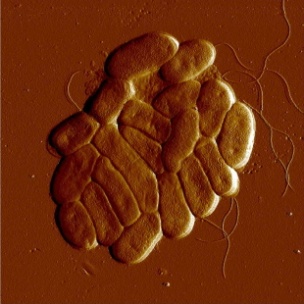

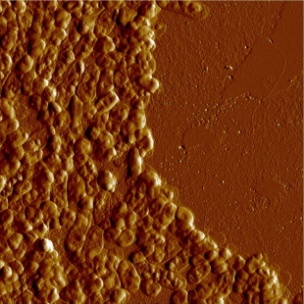


10 min

12 h

24 h

48 h

**Coverslips**

**5 μm**

**5 μm**

**2 μm**

**5 μm**


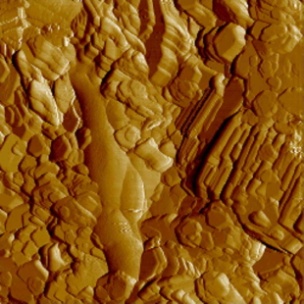


12 h

**Kaolinite**

**1 μm**


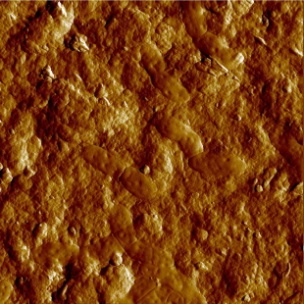

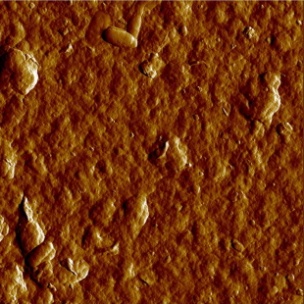

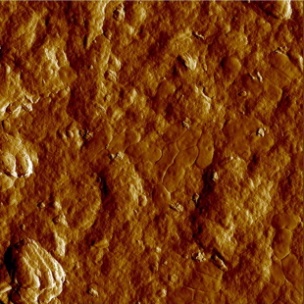

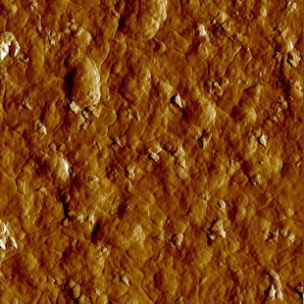


10 min

12 h

24 h

48 h

**Montmorillonite**

**5 μm**

**5 μm**

**5 μm**

**5 μm**


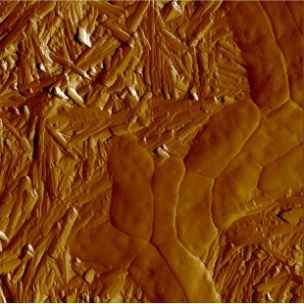

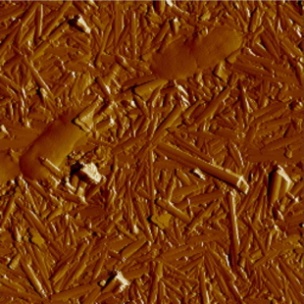

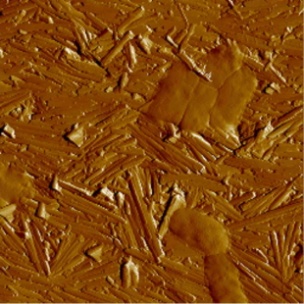

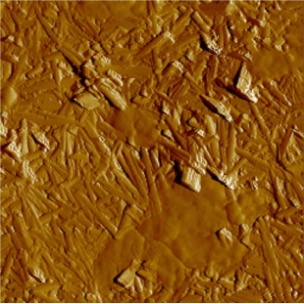


10 min

12 h

24 h

48 h

**Goethite**

**2 μm**

**2 μm**

**2 μm**

**2 μm**

**Fig. S3.** AFM peak force error images of *P. putida* biofilms in LB medium.


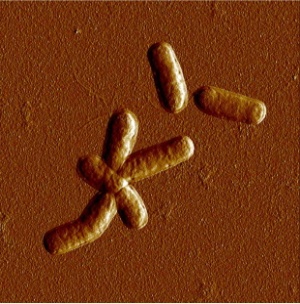

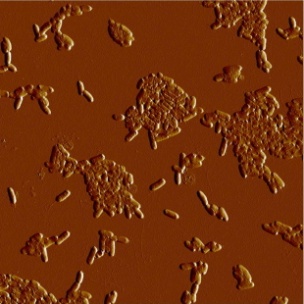

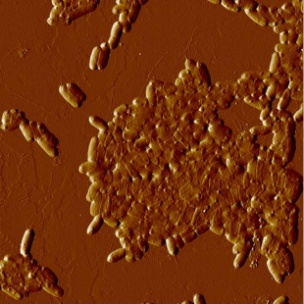

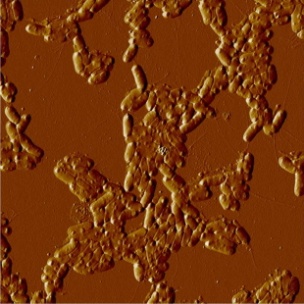


10 min

24 h

48 h

72 h

**Coverslips**

**10 μm**

**2 μm**

**10 μm**

**10 μm**


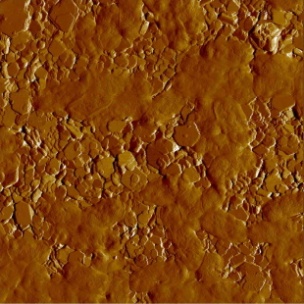


**Kaolinite**

48 h

**2 μm**


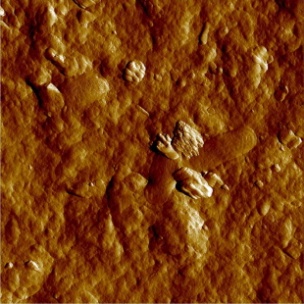

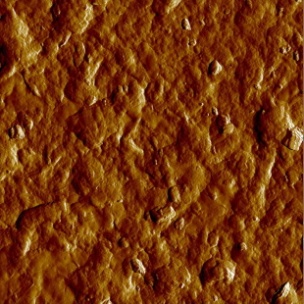

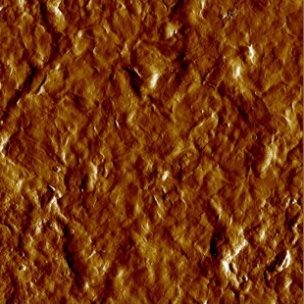


10 min

24 h

48 h

**Montmorillonite**

**2 μm**

**5 μm**

**5 μm**


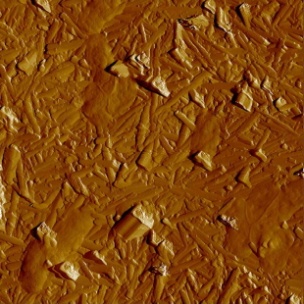

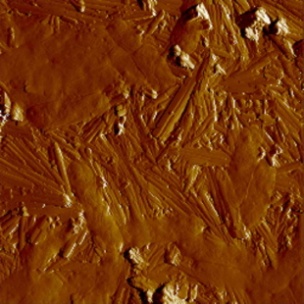

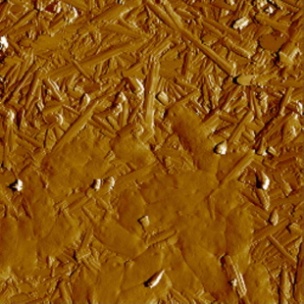

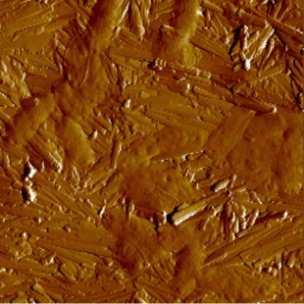


10 min

24 h

48 h

72 h

**Goethite**

**2 μm**

**2 μm**

**2 μm**

**2 μm**

**Fig. S4.** AFM peak force error images of *A. tumefaciens* biofilms in M9 medium.


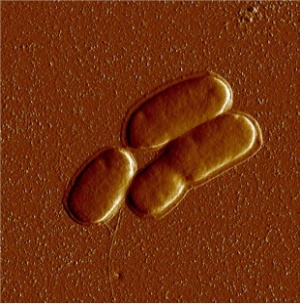

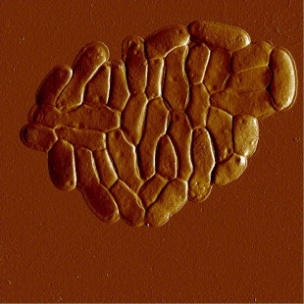

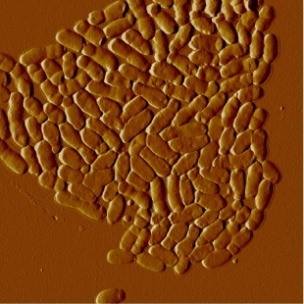

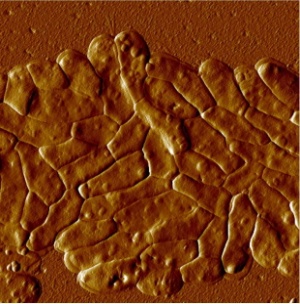


10 min

12 h

24 h

48 h

**Coverslips**

**2 μm**

**4 μm**

**5 μm**

**2 μm**


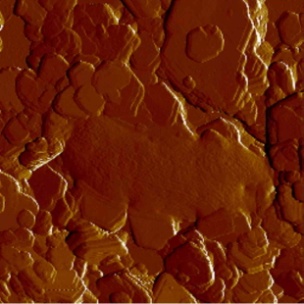


**Kaolinite**

24 h

**1 μm**


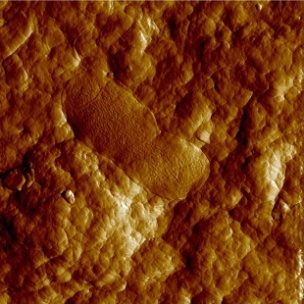

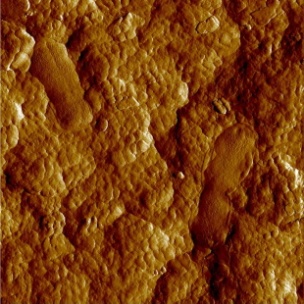

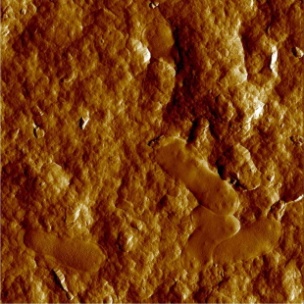

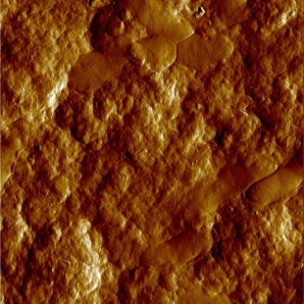


10 min

12 h

24 h

48 h

**Montmorillonite**

**1 μm**

**2 μm**

**2 μm**

**2 μm**


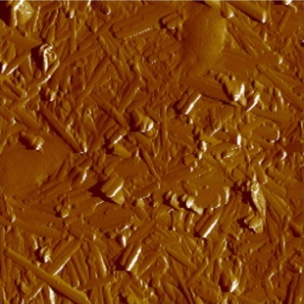

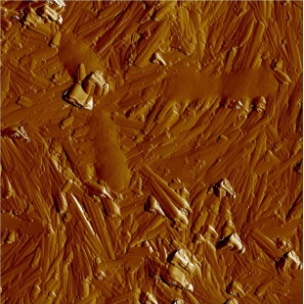

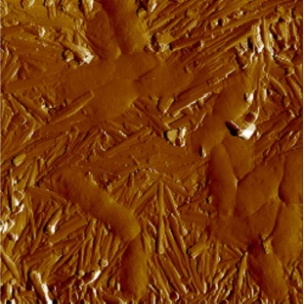

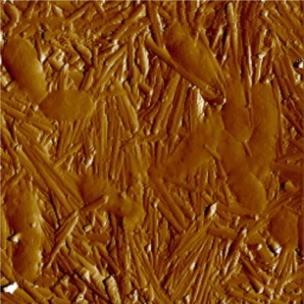


10 min

12 h

24 h

48 h

**Goethite**

**2 μm**

**2 μm**

**2 μm**

**2 μm**

**Fig. S5.** AFM peak force error images of *A. tumefaciens* biofilms in YEB medium.


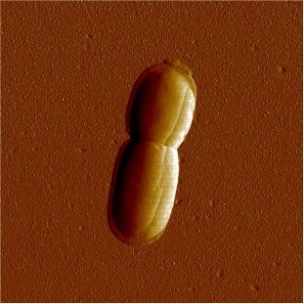

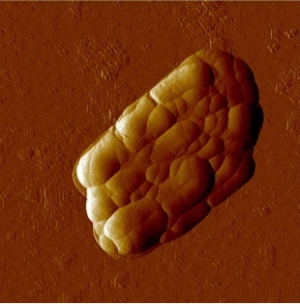

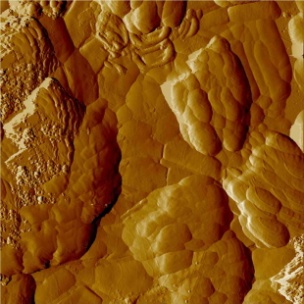

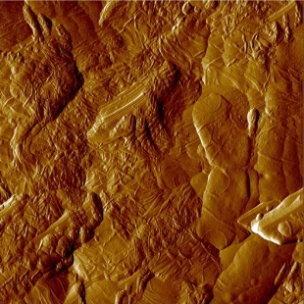


10 min

24 h

48 h

72 h

**Coverslips**

**2 μm**

**2 μm**

**2 μm**

**2 μm**


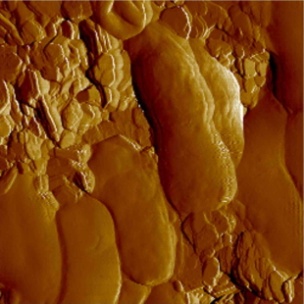

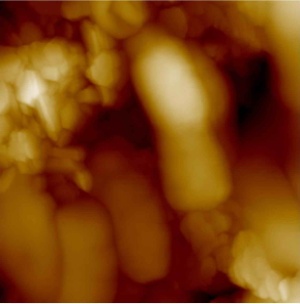

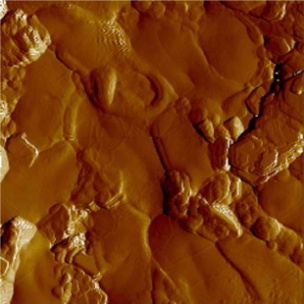

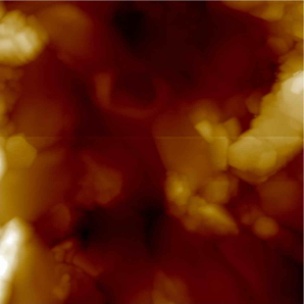


**Kaolinite**

48 h

72 h

**1 μm**

**2 μm**


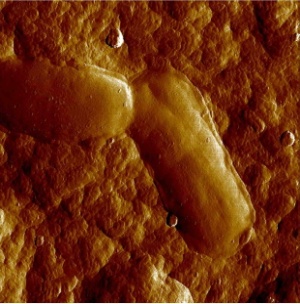

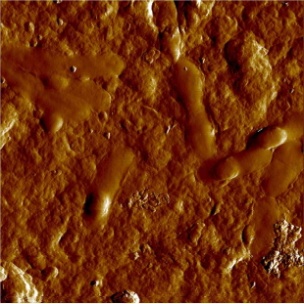

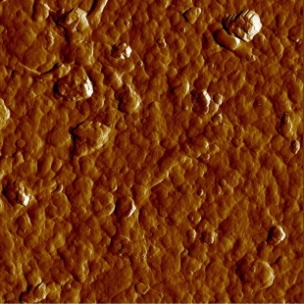

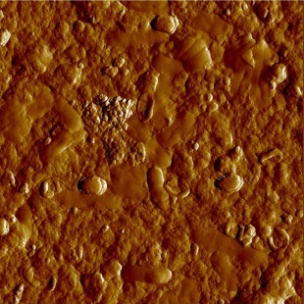


10 min

24 h

48 h

72 h

**Montmorillonite**

**1 μm**

**5 μm**

**4 μm**

**5 μm**


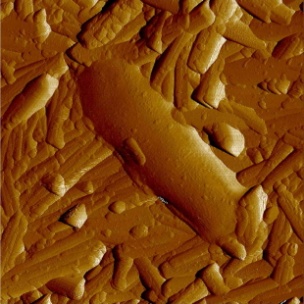

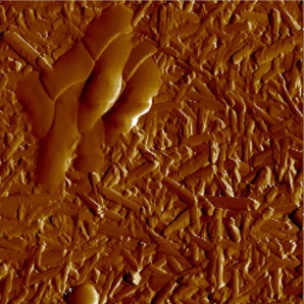

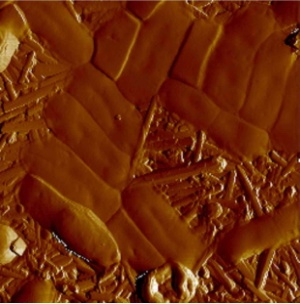

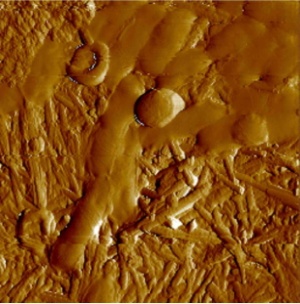


10 min

48 h

72 h

**Goethite**

**1 μm**

**2 μm**

**2 μm**

**2 μm**

**Fig. S6.** AFM peak force error images of *B. subtilis* biofilms in M9 medium.


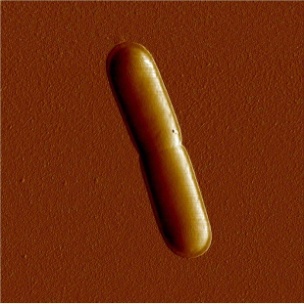

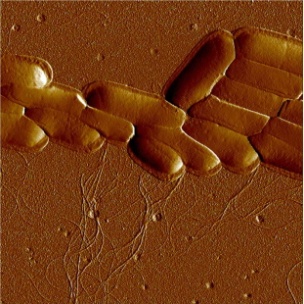

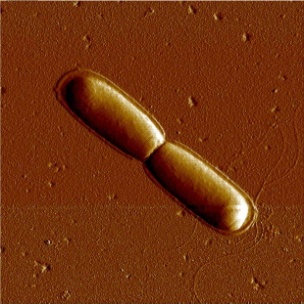

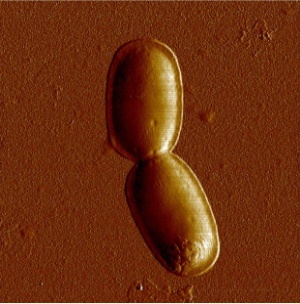


10 min

12 h

24 h

48 h

**Coverslips**

**2 μm**

**2 μm**

**2 μm**

**1 μm**


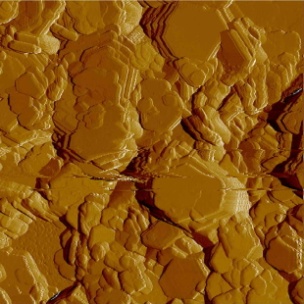


**Kaolinite**

48 h

**1 μm**


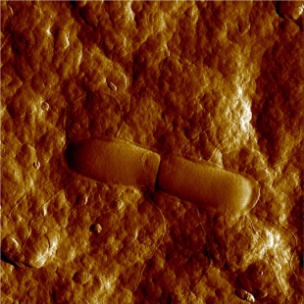

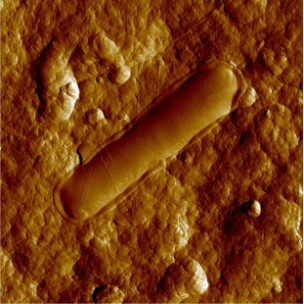

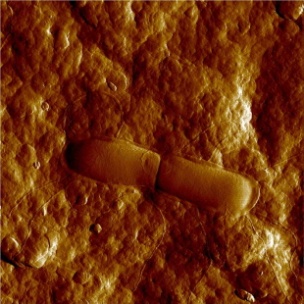

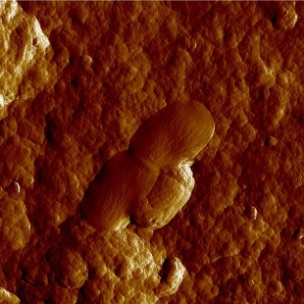


10 min

12 h

24 h

48 h

**Montmorillonite**

**2 μm**

**1 μm**

**2 μm**

**1 μm**


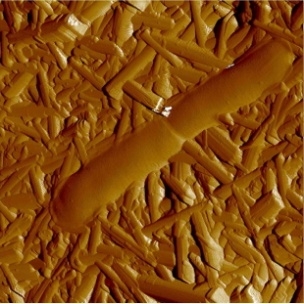

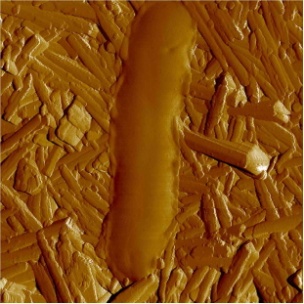

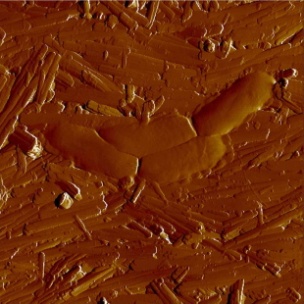

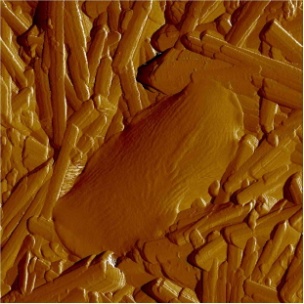


10 min

12 h

24 h

48 h

**Goethite**

**1 μm**

**2 μm**

**1 μm**

**2 μm**

**Fig. S7.** AFM peak force error images of *B. subtilis* biofilms in LB medium.

**Additional experimental procedures**

*Calculation of bacteria-mineral interaction energy profiles*

Derjaguin-Landau-Verwey-Overbeek (DLVO) theory was used to calculate the bacteria-mineral interaction energies as a function of separation distance. Total interaction energies were quantified as the sum of van der Waals and electrostatic interactions and estimated by considering the system using the sphere-sphere model. The retarded van der Waals attractive interaction energy for the sphere-sphere (Φ*_VDW_*) system was calculated with the following expression (Gregory, 1981)

$$\Phi_{VDW}=-\frac{Aa_{p1}a_{p2}}{6h(a_{p1}+a_{p2})}\left[1-\frac{5.32h}{\lambda}\ln\left(1+\frac{\lambda}{5.32h}\right)\right]$$

where A, a_P1_, a_P2_ and h are the Hamaker constant, the cell radius, the mineral radius (Table S1), and separation distance, respectively. λ is the characteristic wavelength (usually considered as 100 nm). The value of A is assumed to be 7.0 × 10^-21^ J for describing bacteria-mineral interactions in water (Hong *et al*., 2012).

The electrical double layer interaction energy for the sphere-sphere (Φ*_EDL_*) system was calculated using the following equation (Hogg *et al*., 1966):

$$\Phi_{EDL}=\frac{\varepsilon_{0}\varepsilon_{r}a_{P1}a_{p2}}{4\left(a_{p1}+a_{p2}\right)}\left\{2\psi_{P}\psi_{C}\ln\left[\frac{1+\exp\left(-\kappa h\right)}{1-\exp\left(-\kappa h\right)}\right]+\left(\psi_{P}^{2}+\psi_{C}^{2}\right)\ln\left[1-\exp\left(-2\kappa h\right)\right]\right\}$$

where ε_0_ denotes the permittivity of vacuum (8.85 × 10^-12^ C V^-1^ m^-1^), and ε_r_ is the relative permittivity of water (80). ψ_P_ and ψ_C_ are the surface potentials of the cell and mineral (V), respectively. The zeta potentials are used as the approximate values instead of the surface potentials (Table S1). κ represents the Debye-Hückel reciprocal length (m^-1^). The value of κ can be calculated by

$$\kappa=\sqrt{\left(\frac{e^{2}\sum n_{i0}z_{i}^{2}}{\varepsilon_{0}\varepsilon_{r}k_{B}T}\right)}$$

where e is the electron charge (1.602 × 10^-19^ C). n_i0_ and z_i_ are number concentration (molecules m^-3^) and valence of ion i in bulk solution, respectively. The ionic strength of the deionized water is estimated to be 0.047 mM. k_B_ is Boltzmann constant (1.38 × 10^-23^ J K^-1^) and T is the absolute temperature of the system (298 K).

*Statistics*

The results are presented as mean ± standard deviations (SD) and analyzed using one-way analysis of variance (ANOVA). Multiple comparisons of mean values were conducted using Fisher’s Least Significance Difference test, with significant differences taken to be those with *p* < 0.05.

**References**

Gregory, J. (1981) Approximate expressions for retard van der Waals interaction. *J Colloid Interf Sci* **83:** 138–145.

Hogg, R., Healy, T.W., and Fuerstenau, D.W. (1966) Mutual coagulation of colloidal dispersions. *Trans Faraday Soc* **62:** 1638−1651.

Hong, Z., Cai, P., Dai, K., Liang, W., Chen, W., and Huang, Q. (2012) Initial adhesion of *Bacillus subtilis* on soil minerals as related to their surface properties. *Eur* *J* *Soil* *Sci* **63:** 457−466.
